# Electrochemically Initiated Synthesis of Polyacrylamide Microgels and Core-shell Particles

**DOI:** 10.1021/acsapm.1c01359

**Published:** 2022-01-05

**Authors:** Nabila Yasmeen, Jakub Kalecki, Pawel Borowicz, Wlodzimierz Kutner, Piyush S. Sharma

**Affiliations:** † Institute of Physical Chemistry, Polish Academy of Sciences, Kasprzaka 44/52, 01-224 Warsaw, Poland; ‡ Faculty of Mathematics and Natural Sciences, School of Sciences, Cardinal Stefan Wyszynski University in Warsaw, Wóycickiego 1/3, 01-938 Warsaw, Poland

**Keywords:** electrochemical polymerization initiation, hydrodynamic electropolymerization, microgel, core-shell particle, inorganic core

## Abstract

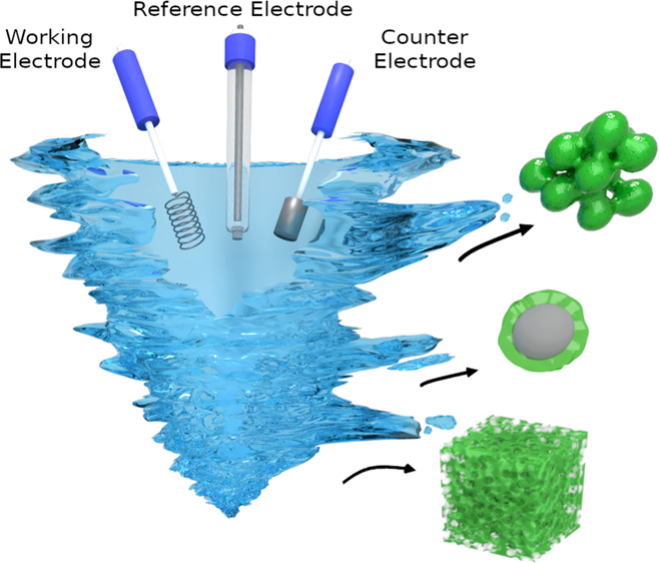

Herein, we developed a simple procedure for synthesizing micrometer-sized microgel particles as a suspension in an aqueous solution and thin films deposited as shells on different inorganic cores. A sufficiently high constant potential was applied to the working electrode to commence the initiator decomposition that resulted in gelation. Under hydrodynamic conditions, this initiation allowed preparing different morphology microgels at room temperature. Importantly, neither heating nor UV-light illumination was needed to initiate the polymerization. Moreover, thin films of the cross-linked gel were anchored on different core substrates, including silica and magnetic nanoparticles. Scanning electron microscopy and transmission electron microscopy imaging confirmed the microgel particles’ and films’ irregular shape and porous structure. Energy-dispersive X-ray spectroscopy indicated that the core coating with the microgel film was successful. Dynamic light scattering measured the micrometer size of gel particles with different combinations of acrylic monomers. Thermogravimetric analysis and the first-derivative thermogravimetric analysis revealed that the microgels’ thermal stability of different compositions was different. Fourier-transform infrared and ^13^C NMR spectroscopy showed successful copolymerization of the main, functional, and cross-linking monomers.

## Introduction

Microgels are cross-linked polymer particles with their size ranging from nanometers to micrometers. These particles can form colloids. Various terms have been used to name a microgel, including the microsphere, microbead, and nanogel.^1^

Polyacrylamide microgels are widely studied among microgels because of their versatile use, including separation,^2,3^ sensing,^4−9^ biomedical applications,^10−13^ and controlled drug release.^14−16^ Moreover, microgels’ ability to absorb solvents of a mass higher than their mass, durability, stimuli-responsiveness, and volume phase transition^12,17−19^ is a property that attracts research interest. One of the most studied polyacrylamide microgels is stimuli-responsive poly(*N*-isopropylacrylamide) (PNIPAM). Generally, PNIPAM microgels are synthesized by copolymerizing the *N*-isopropylacrylamide (NIPAM) monomer with the *N*,*N*′-methylenebisacrylamide (BIS) cross-linking monomer.^9^

Several detailed synthesis procedures have been developed to prepare PNIPAM microgels.^20−23^ The most common procedures include precipitation,^21,24,25^ emulsion,^20,23,26^ and surfactant-free emulsion polymerization.^22,27,28^ Typically, this polymerization involves heating of NIPAM alone or a mixture of NIPAM, BIS, and an additional monomer in an aqueous solution at a slightly elevated temperature in the presence of a water-soluble ammonium peroxydisulfate (APS) initiator. The microgel is formed if the temperature of the solution for polymerization rises above 60 °C.^8,29−31^ Another procedure includes reversible deactivation radical polymerization.^32^

In all of these traditional procedures, polymerization is either photo- or thermally initiated. Moreover, commercial biodegradable surfactants are added to increase the stability and influence the size and morphology of the gel microparticles.^33,34^ However, incomplete removal of the surfactant from the reaction mixture after completion of the reaction results in unavoidable polymer contamination with this surfactant. Moreover, this surfactant changes the physio-chemical properties of the resulting microgel.^1^ Precipitation polymerization offers numerous advantages, including very low polydispersity and the ability to control parameters, such as the particle size, charge, and cross-linking density. However, polymerization at elevated temperatures is unsuitable for encapsulating delicate cargos, such as enzymes, cells, antibodies, and thermally unstable drugs.

Besides, much more straightforward methods for preparing microgels have been reported.^16,35−41^ These methods proposed electrochemically initiated deposition of thin polymer coatings directly on the conducting metal substrates. Interestingly, gel films deposited that way are employed as matrices to suspend other components, for instance, metal nanoparticles and charged species.^39,41^ The introduction of such additional components into the gel network provided materials with broader applications. However, direct electrochemically initiated preparation of a microgel in a solution has not yet been explored.^42,43^

Forced convection is used in electrochemistry to limit diffusive transport within the immediate vicinity of the working electrode, resulting in elevated sensitivity and steady-state mass transport.^44^ This convection can be enforced by magnetically stirring the solution or rotating the electrode. These hydrodynamic techniques facilitate in situ potentiostatic continuous measurements and prevent gel film deposition on electrodes, guaranteeing homogeneous kinetics in the assay volume.

Herein, a facile method for micrometer-sized gel particles preparation is reported. Under hydrodynamic conditions, the electrochemically initiated polymerization facilitated the microgel preparation in an aqueous solution at room temperature. Moreover, altering the pH of the reaction solution generated a stable microgel colloid. These reaction conditions allowed preparing different morphology microgels. For gelation, the main and cross-linking monomers were used. Free-radical generation by applying a suitable constant potential initiated the polymer chain growth without any additives that could be harmful to the resulting microgel’s possible future biomedical applications. NIPAM, methacrylic acid (MA), and BIS were selected as the main and cross-linking monomers for the electrochemically initiated microgel preparation in the presence and absence of core supports.

So far, the microgel preparation under electrochemical hydrodynamic conditions has not been studied. Compared to other microgel preparation procedures, the electrochemically initiated microgel preparation is simple to perform and control.^10,37,45^ More importantly, it requires neither heating nor UV-light illumination to initiate the polymerization, and it can be performed at room temperature. A sufficiently high constant potential applied to the working electrode can initiate the gelation.^37,40,41^ Like other gelation methods, electrochemical gelation is also a free-radical cross-linking polymerization, commencing after the initiator decomposition.^38^ Moreover, a gel film was grafted in the present study over the iron oxide magnetic nanoparticles (MNPs) and silica nanobeads via electrochemically induced polymerization. The main advantage of this approach is the deposition of a gel thin film of different compositions in a single step. The morphology of the microgel and gel film-coated core surfaces has been characterized by scanning electron microscopy (SEM), transmission electron microscopy (TEM), and dynamic light scattering (DLS). The compositional and structural features of the microgels were revealed by thermogravimetric analysis (TGA), first derivative thermogravimetric analysis (DTGA), as well as energy-dispersive X-ray (EDX) spectroscopy, Fourier-transform infrared (FTIR) spectroscopy, and ^13^C NMR spectroscopy.

## Experimental Section

### Materials

NIPAM, MA, BIS, and 50–100 nm size iron oxide MNPs were purchased from Sigma-Aldrich and were used as received. 500 nm size silica beads were procured from Fiber Optic Centre. Deionized Ultrapure Merck Millipore Milli-Q water (18.2 MΩ cm) was used to prepare all aqueous solutions.

### Electrochemical Measurements

An SP-300 BioLogic potentiostat was used for potentiostatic electropolymerization and cyclic voltammetry (CV) measurements. The potentiostat was controlled by EC-Lab BioLogic software. Electrochemical experiments were carried out using a homemade conically shaped three-neck glass cell with a volume of ∼30 mL. A 1 mm diameter Pt wire, an Ag|AgCl electrode, and a seamless Pt cylinder electrode were, respectively, used as the working, reference, and auxiliary electrodes.

### SEM Imaging

The microgel was imaged with SEM using a Nova NanoSEM 450 microscope of the FEI Nova. Microgel samples were first dispersed in an aqueous solution and then drop-cast onto Au film-layered glass slides for imaging.

### Scanning Transmission Electron Microscopy Imaging

Samples for the scanning transmission electron microscopy (STEM) analysis were prepared by dropping the microgel colloid on an amorphous carbon film supported on a 300-mesh copper grid. STEM imaging was conducted on an FEI Talos F200X transmission microscope at 200 kV. The measurements were performed in the scanning (STEM) mode using a high-angle annular dark-field detector. In addition, EDX spectroscopy experiments on a Bruker BD4 instrument were carried out to map the sample’s elemental distribution.

### FTIR Spectroscopy Measurements

Infrared (IR) spectra were recorded with a Vertex 80v FTIR spectrometer using a one-reflection attenuated total reflection computer-controlled Bruker spectrometer equipped with Opus 6.5 software from the same manufacturer. Spectra were recorded with a 2 cm^–1^ resolution. For each spectrum, 1024 scans were acquired. Measurements were performed under decreased (6 hPa) pressure.

### DLS Experiments

The DLS measurements of hydrodynamic particle diameter were carried out using a Zetasizer NS instrument (Malvern Instruments Ltd.). The microgel suspension (0.1%, *w*/*v*) was prepared using Milli-Q water (pH = 7.4) and then sonicated for 15 min before the DLS analysis. To avoid concentration-dependent effects (i.e., particle interactions, etc.), 0.1% *w*/*v* concentration was chosen, as recommended in Zetasizer Nano User Manual for relatively bigger-sized particles. The measurements were performed in triplicate for each sample, and the mean size value, *Z*_avg_, was reported.

### TGA Experiments

The thermal stability of microgels was investigated using a Mettler Toledo TGA/DSC1 thermogravimetric analyzer. The microgel samples were first dried in a vacuum at room temperature for 24 h. Next, an alumina crucible was used to place samples in the furnace. During measurements, samples were purged with nitrogen at a flow rate of 10 mL min^–1^ and heated from room temperature to 1000 °C at a constant temperature gradient of 10 °C min^–1^. The sample weight varied between 5 and 10 mg.

### Electrochemically Initiated Synthesis of Gel Microparticles and Films

The microgel was formed after electrochemical initiation using a homemade electrochemical cell. Typically, a solution of 25 mM MA, 25 mM NIPAM, 25 mM APS, and 50 mM BIS was used for polymerization. Moreover, the monomers were used in other combinations of composition and concentration. However, the total concentration of the monomers was kept at 100 mM for all combinations. Sufficiently high conductivity of the solution was afforded with the 0.1 M KNO_3_ supporting electrolyte. Before electrochemically initiating the polymerization, the solution was deoxygenated with the 20 min argon purge. Furthermore, during all experiments, argon was continuously purged through the solution. Monomers of a similar combination were polymerized in a neutral solution. The solution pH was adjusted with NaOH.

For the electrosynthesis of the gel films grafted MNPs, 2.5 mL of 3 mg mL^–1^ dispersion of MNPs was added to a 25 mL sample of the solution for polymerization, which was 25 mM in MA, 25 mM in NIPAM, and 50 mM in BIS.

Before synthesizing a microgel film, silica nanobeads with an average diameter of 500 nm were first modified using 3-(trimethoxysilyl)propyl methacrylate to obtain the acrylic functionalized silica nanoparticles (NPs).^46^ For that, ethanol (15 mL) and ammonia (1.5 mL) solutions were placed in a round bottom flask (100 mL) connected with a refluxing system. After adding 3-(trimethoxysilyl)propyl methacrylate (10 μL) and silica NPs (400 mg), the system was refluxed at 90 °C overnight to ensure the covalent attachment of the coupling agent to the surface of the silica particles. After modification, the resulting modified silica NPs were suspended in anhydrous ethanol by vigorous shaking in an ultra-sonication bath (10 min, room temperature). After dispersion, silica NPs were centrifuged at 10 000 rpm for 10 min, and the supernatant solution was removed. This procedure was repeated five times for NP purification and dehydration and then those were stored in fresh ethanol (2.5 mL). Subsequently, a 0.5 mL sample of this suspension was added to the 20 mL sample of the solution for electrochemically aided polymerization.

Before starting the electrochemically initiated microgel preparation, the electrochemical properties of the 25 mM MA, 25 mM NIPAM, 25 mM APS, 50 mM BIS, and 0.1 M KNO_3_ solution were examined under an oxygen-free atmosphere. The working electrode potential was linearly cycled between 0 and -1.40 V versus Ag quasi-reference electrode at a scan rate of 50 mV s^–1^. An irreversible cathodic CV peak appeared at ∼0.80 V versus Ag quasi-reference electrode (Figure S1a in the Supporting Information). It corresponded to peroxydisulfate electroreduction.^37^ In effect, soluble persulfate free radicals were formed at the electrode surface. These radicals initiated the polymer chain growth upon interaction with the monomers present in the solution. A pronounced cathodic current at very high negative potentials is due to hydrogen evolution (Figure S1a in Supporting Information). After determining the peroxydisulfate decomposition potential, electrochemical conditions of the microgel synthesis were established. Accordingly, the working electrode potential was kept constant at −0.60 V versus Ag quasi-reference electrode for 3 h with continuous argon purging under hydrodynamic conditions (Figure S1b in Supporting Information).

**Figure 1:**
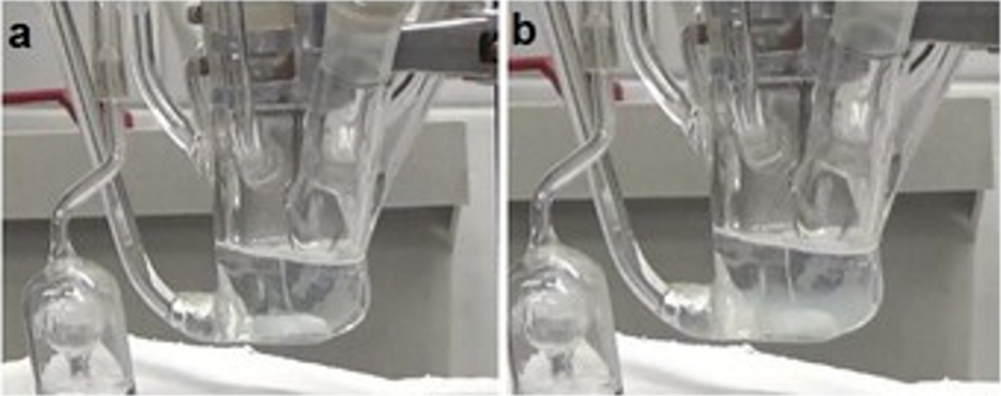
Optical photos of the electrochemical cell (a) just after stopping electrochemical initiation of the polymerization and (b) after 30 min of gelation.

The potentiostatic experiment was performed for 3 h, and then the microgel particles were collected after 30 min (Figure 1). Figure 1 presents the electrochemical cell’s optical images immediately after stopping the electrochemical initiation (Figure 1a) and after 30 min of gelation (Figure 1b). Next, the microgel particles or the core support NPs with microgel shell films were centrifuged at 20 000 rpm for 20 min. This centrifugation was repeated thrice for the complete removal of unreacted substrates. Finally, the microgels were stored in Milli-Q water for further characterizations. Notably, the left overnight solution for polymerization showed no gelation without electrochemical initiation.

## Results and Discussion

Polyacrylamide microgels were prepared as particle suspensions in aqueous solutions and thin films grafted over silica NPs and MNPs. Then, they were extensively characterized.

### Electrochemical Synthesis of Microgel Particles

CV experiments demonstrated APS electroreduction on the Pt disk working electrode starting from −0.30 V versus Ag quasi-reference electrode (Figure S1a in Supporting Information). This electroreduction resulted in soluble persulfate free radicals at the electrode surface. Under solution agitation conditions, the produced radicals can escape from the electrode surface to the solution bulk.^16,36,37^ Herein, this escaping was enhanced by vigorous magnetic stirring of the solution. At potentials more negative than −1.0 V versus Ag quasi-reference electrode, acrylic monomers can be electroreduced. However, this electroreduction can generate an undesired dimeric acrylic product.^45^ Therefore, a relatively low constant potential of −0.60 V versus Ag quasi-reference electrode was selected to generate free radicals by decomposing APS. A corresponding potentiostatic curve is presented in Figure S1b in the Supporting Information. The current reached its constant minimum value after 30 min. Here, the persulfate concentration in the polymerization solution was higher than that reported earlier.^37^

**Scheme 1:**
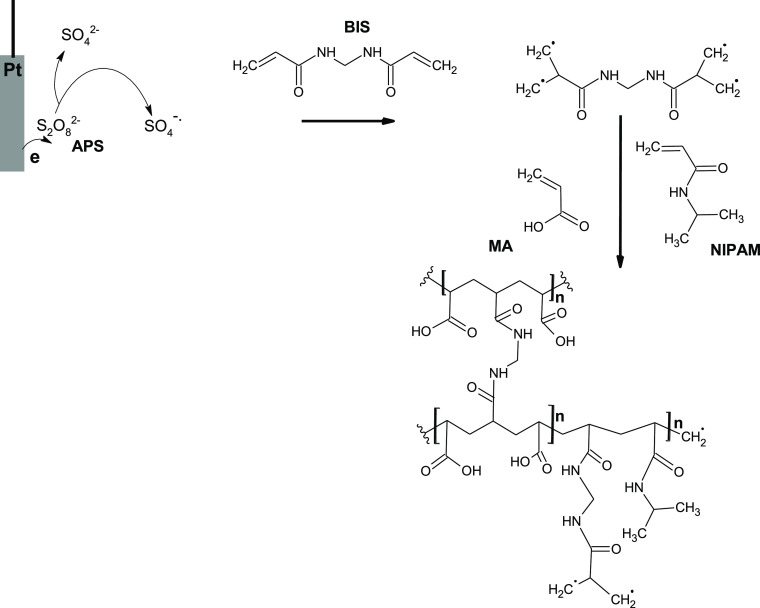
Mechanism of Electrochemically Initiated Microgel Synthesis

The water-soluble NIPAM monomer polymerization mechanism has widely been studied.^1,10,37^ Similarly, as in the previously described syntheses, in our electrochemically initiated polymerization, the solution for polymerization was initially homogeneous (Scheme 1). As the potentiostatic synthesis proceeded, more and more APS was cleaved, and the amount of water-soluble free radicals became sufficient for initiating the polymerization (Scheme 1).^47^ First, these radicals attacked the BIS monomer.^48^ The kinetic studies of BIS and NIPAM copolymerization indicated that the BIS monomer reacts faster than the NIPAM monomer, despite the latter being hydrophilic.^49^ The BIS redox-active center targeted the NIPAM and MA monomer double bonds to result in more extended oligomers. Ultimately, these oligomers combine and nucleate to form microgel particles. A suitable amount of the cross-linking monomer in the solution promotes entropic precipitation.^50,51^

Moreover, this NIPAM and MA monomer incorporation in the growing polymer chain substantially decreases the polymer’s solubility. Usually, density of no part in the microgel structure is homogeneous. This lack of homogeneity arises from the different reactivity of the main and cross-linking monomers.^15^ The faster polymerizing monomers generate centers of higher density in the polymer interior, while a fuzzy surface with dangling chains constitutes its outer part. However, at a considerably high concentration of a cross-linking monomer, coverage of carboxyl terminated MA chains can be high, and the number of reactive sites can be sufficient.^52^ Thus, Scheme 1 proposes a tentative mechanism of electrochemically initiated microgel synthesis.

### SEM and TEM Characterization of the Morphology of Microgel Particles

**Figure 2:**
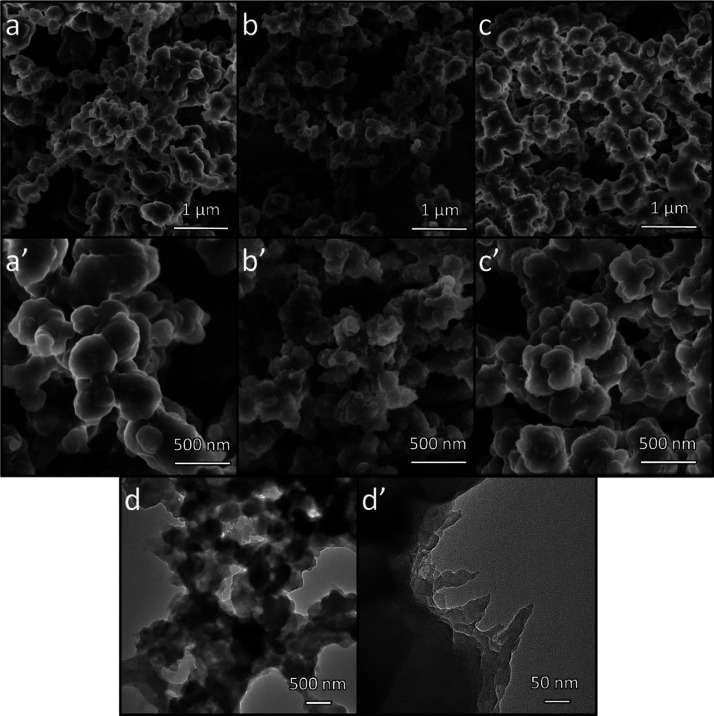
SEM images of the microgels prepared with monomer combinations of (a,a′) NIPAM-BIS, (b,b′) MA-BIS, and (c,c′) NIPAM-MA-BIS. (d,d′) STEM image of the NIPAM-MA-BIS microgel.

The microgels, prepared with all monomer combinations, were imaged with SEM and STEM to study their morphology (Figure 2). The images revealed that the microgel particles were mainly irregular. Interestingly, the size of the microgel particles, prepared by combining NIPAM and BIS (Figure 2a,a′), appeared similar to that by combining NIPAM, MA, and BIS (Figure 2c,c′). A closer examination of the images unraveled a globular shape of the NIPAM-BIS microgel particles (Figure 2a,a′).

The MA-BIS particles were smaller (Figure 2b,b′). The irregular shape of NIPAM-MA-BIS particles presumably resulted from the aggregation of smaller particles (Figure 2c,c′). Edges of these particles are seen. This high aggregation likely arises from thorough drying of the microgel during SEM imaging. Moreover, the van der Waals and hydrophobic interactions might contribute to this aggregation.

Presumably, the MA monomer incorporation in the microgel aided in generating globular microgel particles. The STEM image of the NIPAM-MA-BIS microgel demonstrates the porosity of the particles with a fuzzy surface (Figure 2d,d′), in accordance with earlier reports.^1,53,54^

Interestingly, the microgel morphology was different if the solution pH for polymerization was higher than the acid’s p*K*_a_ (Figure S2 in the Supporting Information). At a high pH, where the acid monomer was negatively charged, the microgel particles were partially interconnected, yielding a soft gel-like soluble material. Consequently, it was difficult to separate this microgel from the aqueous solution. In contrast, the uncharged microgel particles were densely packed when polymerization was performed at a low solution pH (Figure 2).

Further, to confirm the porosity and determine the microgel surface area, the Brunauer–Emmett–Teller (BET) analysis was performed (Figure S3a in Supporting Information). The adsorption isotherms constructed showed a steep increase in the adsorbed volume, typical of IUPAC type IV isotherms. The pore size distribution, calculated by the Barrett–Joyner–Halenda method, varied between 2.5 and 15 nm with a maximum centered at 5 nm (Figure S3b in the Supporting Information). The surface area of the NIPAM-MA-BIS microgel was relatively high, equaling 136 m^2^ g^–1^.

### FTIR and ^13^C NMR Spectroscopy Characterization of Microgel Particles

**Figure 3:**
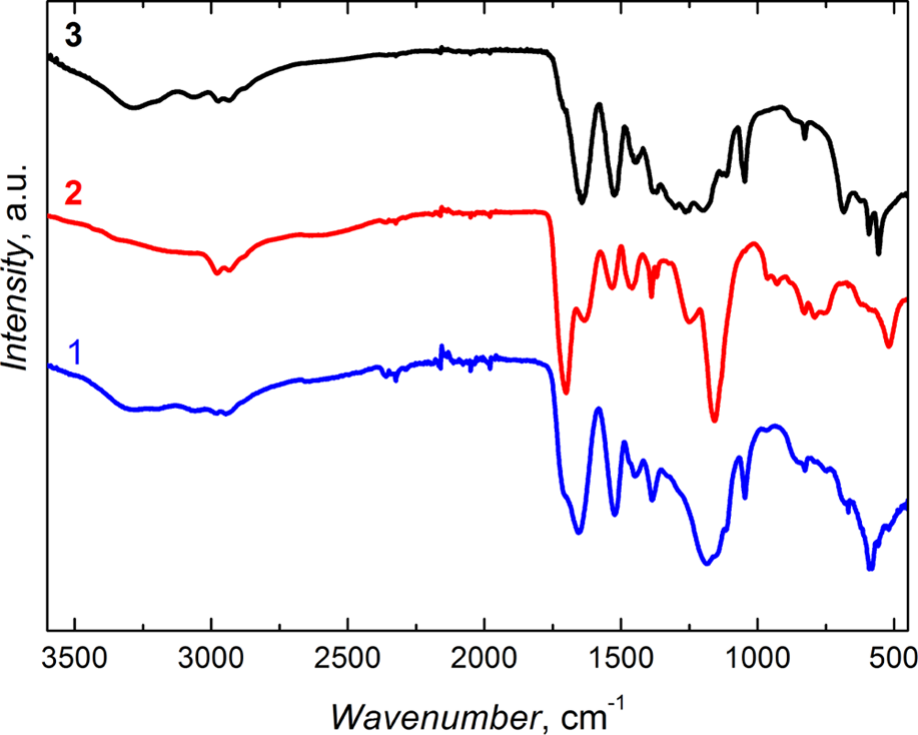
FTIR transmission spectra of microgels prepared by electrochemically initiated copolymerization of NIPAM, MA, and BIS with the combinations of (1) NIPAM-MA, (2) NIPAM-MA-BIS, and (3) NIPAM-BIS.

FTIR transmission spectroscopy was applied to confirm electrochemically initiated polymerization. The structural features of the microgel particles, prepared by polymerizing the NIPAM, MA, and BIS monomers with different combinations, were examined by FTIR spectroscopy (Figure 3). The MA monomer incorporation in the polymer manifested itself by a sharp band at ∼2990 cm^–1^ (spectra 1 and 2 in Figure 3). This band is characteristic of the stretching vibration of the–OH bond. The band at ∼2930 cm^–1^ corresponds to the stretching vibration of the C–H bond of the −CH_3_ substituent. It is present in the spectra of all microgel particles (spectra 1–3 in Figure 3).^55^ The band at ∼3300 cm^–1^ corresponds to the stretching vibration of the N–H bond. This band is pronounced in spectra 1 and 3, and it is barely seen in spectrum 2 in Figure 3.

The spectrum of the copolymer, prepared with NIPAM, MA, and BIS, contained all the stretching vibration bands (Figure 3). The band at 1550 cm^–1^, assigned to the N–H bond bending vibration, was present in the spectra of all microgels (Figure 3). The band of the C–H bending vibration of the −CH_3_ substituent appeared at 1390 cm^–1^ (Figure 3). For the isopropyl substituent, this C–H band was characteristically split into two bands, one at 1388 and the other at 1370 cm^–1^ (spectrum 2 in Figure 3). Moreover, this band appeared in the NIPAM, MA, and BIS copolymer spectra at similar wavenumbers. However, it was relatively less intense (spectrum 3 in Figure 3). Sharp bands between 1630 and 1650 cm^–1^ confirm the carbonyl group presence in all polymers (Figure 3). The band at ∼1700 cm^–1^, associated with the stretching vibration of the MA monomer′s carbonyl bond, appeared as a shoulder in spectrum 1, but it was quite intense in spectrum 2 in Figure 3.

**Figure 4:**
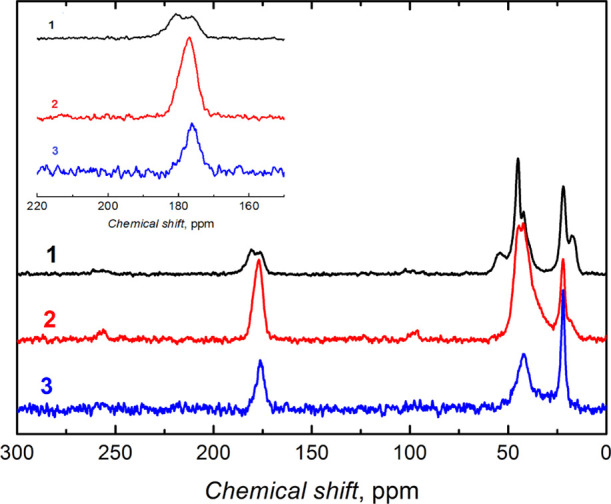
^13^C NMR spectra of three different microgel particles prepared by electrochemically initiated copolymerization of NIPAM, MA, and BIS with the combinations of (1) NIPAM-MA, (2) NIPAM-MA-BIS, and (3) NIPAM-BIS. Inset shows the magnified spectra in the chemical shift range of 150–220 ppm.

Moreover, the microgel structural features were disclosed with the ^13^C NMR spectroscopy measurements (Figure 4). The microgel particles prepared by copolymerizing NIPAM and MA reveal a peak at ∼178 ppm typical of the amide carbon of NIPAM (Figure 4). The peak at ∼181 ppm confirms the presence of the −C=O bond of the MA moiety in the copolymer (spectrum 1 in Figure 4). The microgel prepared by combining all monomers shows a broad peak between 175 and 180 ppm, thus indicating an overlap of the −C=O and −C–NH carbon peaks (spectrum 2 in Figure 4). Worth mentioning, this peak was absent for the microgel particles not containing MA (spectrum 3 in the inset to Figure 4). Peaks at 44 and 21 ppm can be attributed to carbon atoms of the isopropyl and −CH_3_– substituents of NIPAM, as indicated in earlier reported spectra for these substituents.^56,57^

FTIR and ^13^C NMR spectroscopy structural analyses confirmed the successful electrochemically initiated copolymerization of monomers with different activities.

### Microgel Particle Analysis with DLS

**Figure 5:**
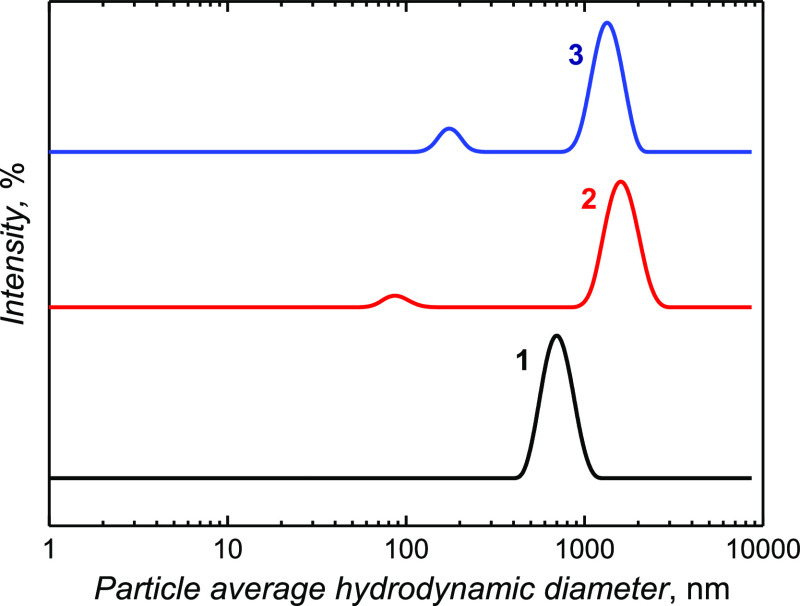
DLS size analyses of microgel particles prepared under hydrodynamic conditions by electrochemically initiated polymerization of (1) NIPAM-MA-BIS, (2) NIPAM-BIS, and (3) NIPAM-MA monomer combinations.

The DLS technique determined the average hydrodynamic diameter of microgel particles (Figure 5). If the particles exhibit random Brownian motion, their diffusion coefficient can readily be determined from the autocorrelation function’s decay (Figure S4 in Supporting Information). The microgel suspensions were diluted before measurements. The dispersity in aqueous solutions of the NIPAM-MA-BIS, NIPAM-BIS, and NIPAM-MA microgels was high. A relatively high polydispersity index, PDI > 0.1, indicated a broad distribution of the particle sizes (Figure 5), well matching the SEM imaging (Figure 2). Due to the anionic APS initiator application in all polymerizations, the zeta-potential of microgels in their swollen state was slightly negative, being −9 to −10 mV. The particle size of the NIPAM-MA-BIS microgel appeared smaller than those of the NIPAM-BIS and NIPAM-MA microgels. Presumably, the incorporation of MA facilitated the formation of a microgel with a defined particle size. However, surprisingly, the particle size of the NIPAM-MA microgel ranged from 1 to 2 μm (curve 3 in Figure 5). The particle size distribution of the microgel without MA, that is, NIPAM-BIS, was broader, ranging from 1 to 2.5 μm (curve 2 in Figure 5). The small peaks in DLS (Figure 5) for NIPAM-BIS and NIPAM-MA monomer combinations correspond to oligomers.^58^

Moreover, the DLS measurements were performed at different time intervals after ceasing electrochemical initiation and hydrodynamic agitation (Figure S5 in the Supporting Information) to investigate the gelation progress with time. For that, the microgel was prepared by combining the NIPAM, MA, and BIS monomers. As a result, the size distribution of microgel particles collected immediately after seizing the electrochemical initiation was broad (curve 1 in Figure S5 in Supporting Information), that is, ranging from 1000 to 4000 nm. This wide distribution can arise from the presence of growing polymer chains.

The size of the microgel particles was further measured after sample collection at 30 min of gelation. This size was remarkably smaller, ranging from 500 to 1000 nm (curve 2 in Figure S5 in Supporting Information). Finally, the size of particles collected after 1 h grew again. This size ranged from 1000 to 5000 nm. This growth in size with the time of microgel preparation can be due to particle aggregation.

According to the above observation, we speculate on the following polymerization mechanism. A water-soluble sulfate radical initiates the formation of BIS radicals. Then, those radicals react with other monomers to grow oligomers in the solution until reaching a critical chain length. Afterward, the growing chain collapses to become an unstable colloidal particle. The precursor particles can follow one of at least two competing routes. For instance, they can deposit onto an existing colloidally stable polymer particle or aggregate with other precursor particles until they form a large particle to be colloidally stable.

### Thermal Stability of Microgel Particles

**Figure 6:**
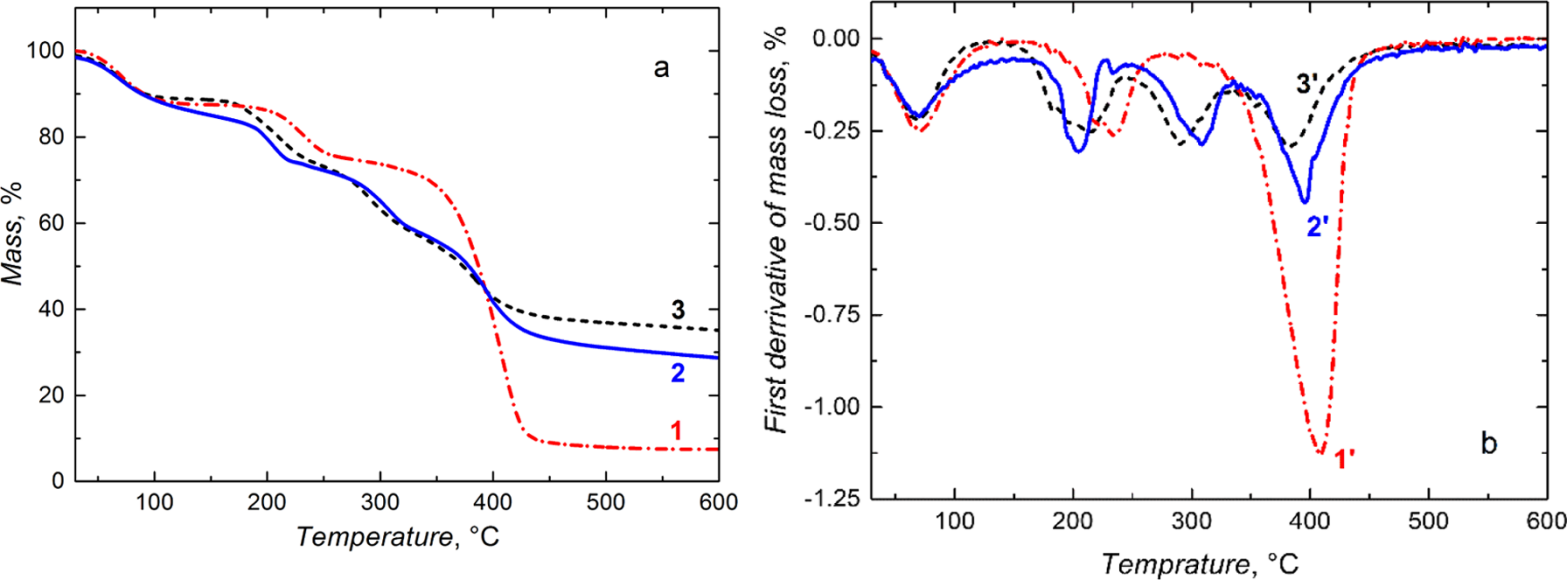
(a) TGA and (b) DTGA thermograms for dried (1,1′) NIPAM-MA, (2,2′) NIPAM-BIS, and (3,3′) NIPAM-MA-BIS microgel particles. The particles were heated up to 1000 °C at a constant rate of 10 °C min^–1^.

The microgel thermal stability was investigated by TGA and the first DTGA. Thermograms in Figure 6a,b present the microgel mass loss in the TGA and DTGA experiments, respectively. Mainly, the DTGA thermograms determined temperatures of the most significant mass losses. The first mass loss of ca. 12–15% at ∼70 °C indicates water removal. The second and the third losses between 200 and 450 °C were caused by the decomposition of the NIPAM-MA polymer backbone (thermogram 1 in Figure 6a). Three corresponding endothermic DTGA peaks (thermogram 1′ in Figure 6a) confirmed the presence of three successive stages in the thermal decomposition of this polymer. The first significant decomposition above ∼200 °C was assigned to the cleavage of easily breakable groups, followed by complete polymer degradation at ∼390 °C. A similar trend was observed for other microgels.

Interestingly, thermograms 2 and 2′ in Figure 6a,b, respectively, for the NIPAM-BIS microgel particles were slightly shifted to a higher temperature of ∼400 °C. The smallest mass loss for the NIPAM-MA-BIS microgel combining all monomers indicated that this microgel was thermally most stable (thermogram 3 in Figure 6a) while the NIPAM-MA was the least stable microgel (thermogram 2 and 2′ in Figure 6a,b, respectively). The decomposition of this microgel involved three steps. Most likely, the absence of any cross-linking monomer was the reason for this microgel’s lower stability. Other microgel particles decomposed via four stages (thermograms 2 and 3 in Figure 6a as well as 2′ and 3′ in Figure 6b).

### Electrochemical Synthesis and Characterization of Microgel Films Grafted over Silica NPs and MNP Cores

Multi-step polymerization conditions have already been applied to coat different inorganic core particles with films of the PNIPAM shell alone or combined with a cross-linking monomer.^14,59−64^ For instance, a PNIPAM shell was grafted over selected cores via multi-step reversible addition–fragmentation transfer polymerization.^62,63^ These hybrid NPs combine the properties of an inorganic core and a microgel shell. As a result, they provide appealing catalytic^65^ and nontoxic drug delivery systems.^66^

Toward that, silica nanobeads and MNPs were herein coated with thin microgel films by electrochemically initiated polymerization. Initially developed for coating MNP cores, this procedure was slightly modified to coat the silica NP cores. For microgel grafting over silica NPs, the pH of the solution for polymerization was adjusted to 7.0. It appeared that at this pH value, a macroporous film was formed. This film was favorable in the coating of nanometer-sized silica particles. Finally, the monomers with different combinations and MNPs were added to the solution for polymerization to prepare the MNPs coated with microgel films. This solution’s pH was not adjusted.

### SEM and STEM Characterization of the Morphology of Microgel Films Grafted over Silica Nanobeads and MNP Cores

**Figure 7:**
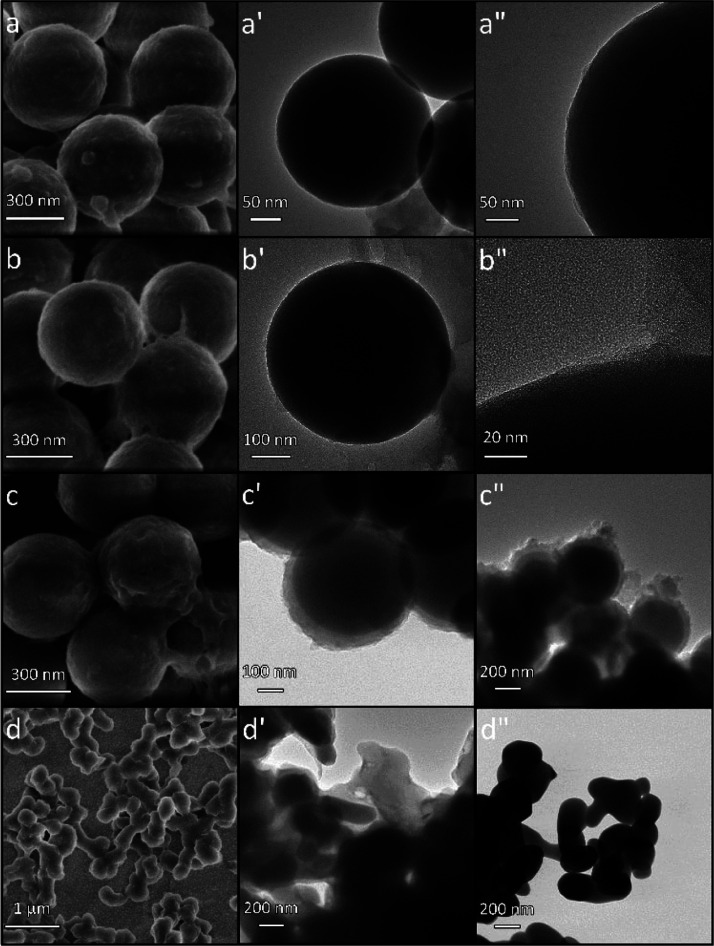
(a–c) SEM and (a′, a″, b′, b″ and c′ and c″) STEM images of microgel films deposited on silica NP cores. The films were prepared by combining different supports and monomers, that is, (a, a′, and a″) (silica NP)-(NIPAM-MA-BIS), (b, b′, and b″) (silica NP)-(NIPAM-BIS), and (c, c′, and c″) (silica NP)-(MA-BIS). The (MNP)-(NIPAM-MA-BIS) image of (d) SEM and (d′) STEM, as well as the (d″) STEM image of bare MNPs.

The SEM image clearly shows that the microgel film with the NIPAM-MA-BIS monomer combination coats the silica nanobead cores (Figure 7a). The STEM image indicates that this film is relatively thin (Figure 7a′,a″). The NIPAM-BIS microgel coating presents itself similarly (Figure 7b,b′,b″). Both SEM and STEM imaging confirmed the formation of a thick microgel film for the MA-BIS combination (Figure 7c,c′,c″, respectively). The SEM and STEM images of NIPAM-MA microgel film-coated silica nanobeads were not seen clearly, suggesting that the film was very thin (Figure S6 in the Supporting Information). The image in Figure 7d shows the MNPs coated with the microgel film with the NIPAM-MA-BIS combination. The STEM image confirms the inclusion of MNPs in the microgel net (Figure 7d′). However, the morphology of film-coated MNPs differed from that of bare MNPs (Figure 7d'').

**Figure 8:**
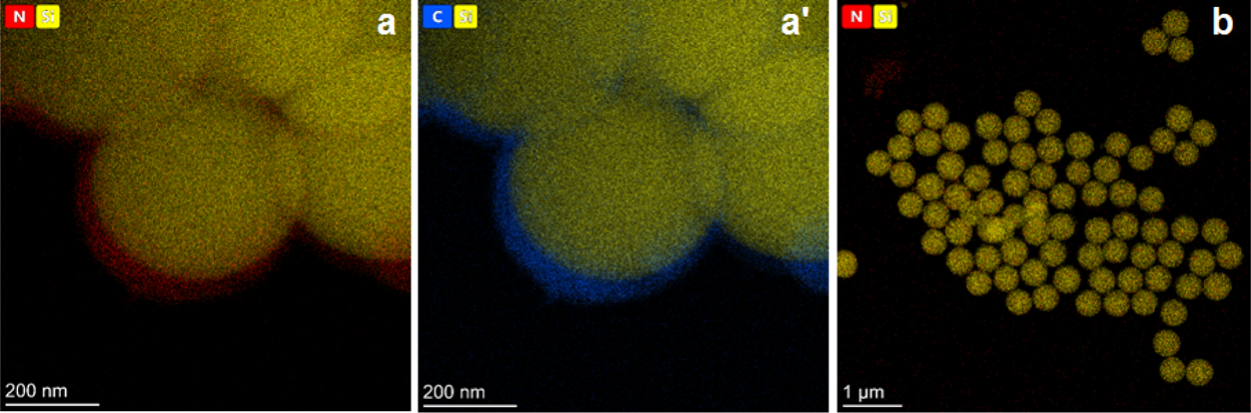
EDX spectroscopy mapping of elemental distribution over the (a,a′) (silica NP)-(MA-BIS) and (b) (silica NP)-(NIPAM-MA) microgel film-coated silica NPs.

EDX spectroscopy mapping indicated the elemental distribution of the (Figure 8a,a′) MA-BIS and (b) NIPAM-MA microgel film-coated silica NPs. Figure 8 shows the elemental maps of representative NPs revealing a core-shell structure of the Si core (olive yellow), as well as the C (blue)- and N (red)-containing microgel shells. This result supports the deposition of over ∼50 nm thick microgel films on the silica nanobead cores (Figure 8a,a′). These results postulated that the reactivity of monomers of different combinations was different. The SEM and TEM imaging revealed that the polymer film shell growth over the core substrate was pronounced in the presence of MA and BIS monomers. With another combination, the films were thinner (Figures 7b,b′, 8b, and S6).

### Thermal Stability of Microgel Films Grafted over Silica Nanobeads and MNP Cores

**Figure 9:**
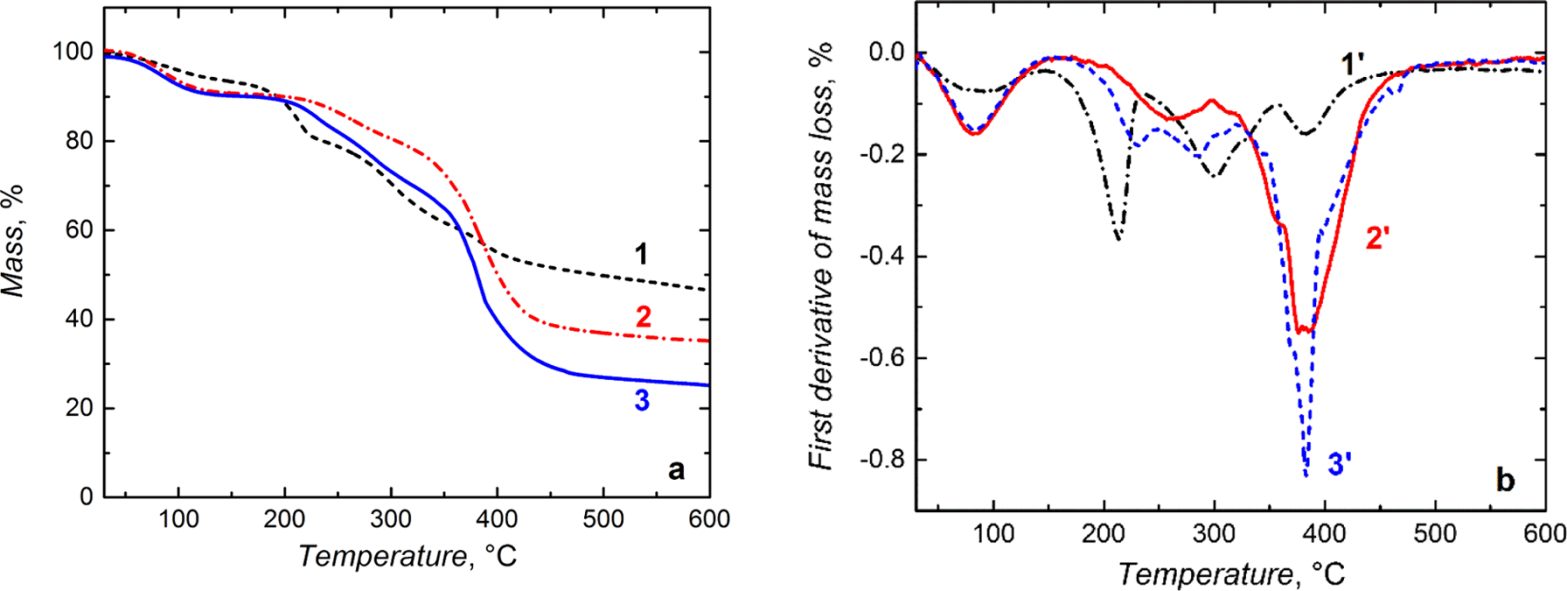
(a) TGA and (b) DTGA thermograms for dried microgel core-shell samples of (1,1′) (silica nanobead)-(NIPAM-BIS), (2,2′) (silica nanobead)-(MA-BIS), and (3,3′) (MNP)-(NIPAM-MA-BIS). The samples were heated up to 1000 °C at a constant rate of 10 °C min^–1^.

Similar to the stability of microgel particles (Figure 6), the stability of the microgel films, deposited on the silica nanobead and MNP cores, was investigated by both TGA and DTGA (Figure 9). The first mass loss, associated with water removal from both the (silica nanobead)-(MA-BIS) and MNP-(NIPAM-MA-BIS) core-shell nanostructures, was ∼10% at ∼110 °C. For (silica NP)-(NIPAM-BIS), this loss was lower, equaling 5%, thus indicating less water content in the shell.

Moreover, the water loss was slower. The second and the third mass loss in the TGA thermogram was in the range of 200–450 °C. They arose from the thermal decomposition of the polymer shell. Interestingly, MNP-(NIPAM-MA-BIS) decomposition (thermogram 3 in Figure 9a) was more extensive than those of silica nanobeads coated with microgel films (thermograms 1 and 2 in Figure 9a), presumably, because of the decomposition of the magnetic core particles above 450 °C for the former. Apparently, the microgel shell deposited on silica nanobead cores by combining the MA and BIS monomers is thermally less stable (thermogram 2 in Figure 9a) than the one prepared by combining the NIPAM and BIS monomers (thermogram 1 in Figure 9a).

### FTIR Spectroscopy Characterization of Microgel Shells

**Figure 10:**
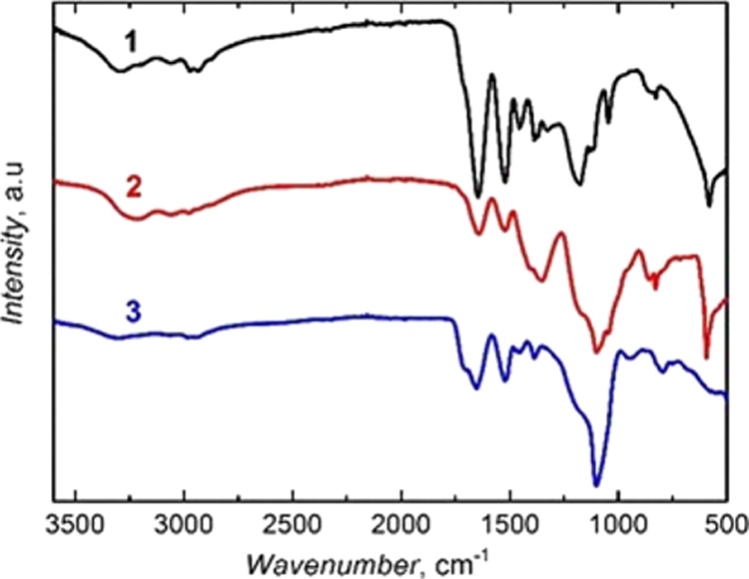
FTIR transmission spectra of thin gel films grafted over inorganic cores at the monomer composition of (1) (silica NP)-(MA-BIS), (2) (silica NP)-(NIPAM-BIS), and (3) (MNP)-(NIPAM-MA-BIS) by electrochemically initiated copolymerization.

The presence of the bands in the FTIR spectrum at ∼1650 and 1520 cm^–1^ corresponding to the C=O bond stretching and N–H bond bending vibration, respectively, of the amide group, confirms the microgel film deposition on the silica NP cores (spectra 1 and 2 in Figure 10), as well as on the MNPs (spectrum 3 in Figure 10). The band appearing as a shoulder at ∼1710 cm^–1^ originates from the stretching vibration of the C=O bond of the carboxyl group in the shell containing the MA moiety (spectrum 1 in Figure 10). The band at 1100 cm^–1^ is related to the Si–O bond stretching vibration (spectra 1 and 2 in Figure 10). All the spectra contain main bands at ∼2930 cm^–1^ assigned to the stretching vibration of the C–H bond of the −CH_3_ substituent (spectra 1–3 in Figure 10). The N–H bond’s stretching vibration appeared as the band at ∼3300 cm^–1^ (spectra 1–3 in Figure 10). Similarly, as above, the presence of a sharp band at ∼2990 cm^–1^ of the –OH bond stretching vibration (spectra 1 and 3 in Figure 10) confirms the MA monomer’s incorporation in the shell. These characteristic bands evidence the successful deposition of the microgel shells with different monomer combinations.

## Conclusions

We successfully exploited electrochemistry under hydrodynamic conditions to pursue an alternative way to prepare different morphology polyacrylamide microgels and core-shell particles. The silica and MNPs used as cores were successfully coated with the microgel films prepared with various monomer combinations. SEM and STEM imaging positively confirmed the syntheses of the microgel particles of different morphologies and thin coatings. The DLS analyses indicated that the microgels were stable in neutral aqueous solutions. The FTIR and ^13^C NMR spectra provided the direct pieces of evidence that MA was successfully copolymerized with the NIPAM and BIS monomers. Moreover, the TGA and DTGA analyses demonstrated that the microgels’ thermal stability was comparable to the stability of the analogous microgels prepared according to literature procedures. A simple greener synthesis procedure developed herein may appear helpful in making more advanced eco-friendly gel structures for biological and industrial applications.

## Supporting Information Available

Electrochemically initiated microgel preparation, electrochemical SEM images of a microgel prepared at pH = 7.4; the BET isotherm of a microgel; the correlation function intensity for microgel particles prepared under hydrodynamic conditions by electrochemically initiated polymerization; pore size distribution of a microgel; microgel particle size, as measured by DLS and correlation function intensity at different time intervals after initiating the polymerization; and the SEM and TEM images of (silica NP)-(NIPAM-MA) (PDF)
