# Supplementary material for: Electrochemically Initiated Synthesis of Polyacrylamide Microgels and Core-shell Particles
Source: ACS Appl Polym Mater. 2022 Jan 5;4(1):452–62. doi: 10.1021/acsapm.1c01359 (PMC8762648; doi:10.1021/acsapm.1c01359)
Supplement: Supplementary file 1 — ap1c01359_si_001.pdf [file ap1c01359_si_001.pdf]

Supporting Information

Electrochemically Initiated Synthesis of Polyacrylamide Microgel and Core-shell Particles

Nabila Yasmeen[1], Jakub Kalecki[1], Pawel Borowicz[1], Wlodzimierz Kutner[1,2], Piyush Sindhu Sharma[1,*]

[1] Institute of Physical Chemistry, Polish Academy of Sciences, Kasprzaka 44/52, 01-224 Warsaw, Poland

[2] Faculty of Mathematics and Natural Sciences. School of Sciences, Cardinal Stefan Wyszynski University in Warsaw, Wóycickiego 1/3, 01-938 Warsaw, Poland

*Corresponding author E-mail address: psharma@ichf.edu.pl (Piyush Sindhu Sharma)

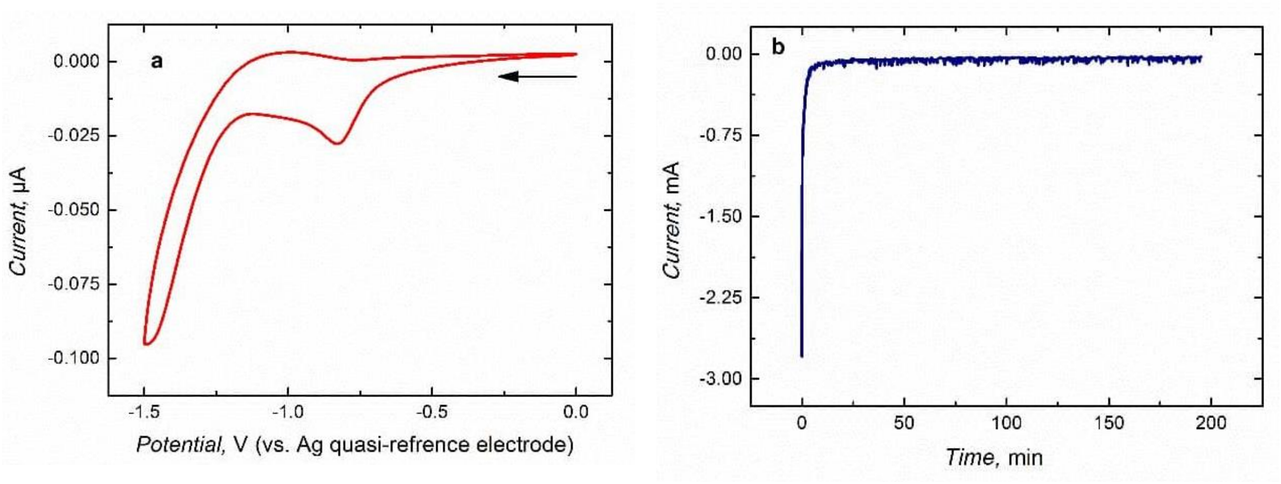

Figure S1. The recorded at the 0.7-mm diameter Pt disk electrode in the solution of 25 mM **MA**, 25 mM **NIPAM**, 50 mM **BIS**, 25 mM **APS**, and 0.1 M in KNO_3 (a) cyclic voltammogram at a scan rate of 50 mV s^{-1} and (b) potentiostatic current transient at -0.60 V vs. Ag quasi-reference electrode under vigorous magnetic stirring conditions.

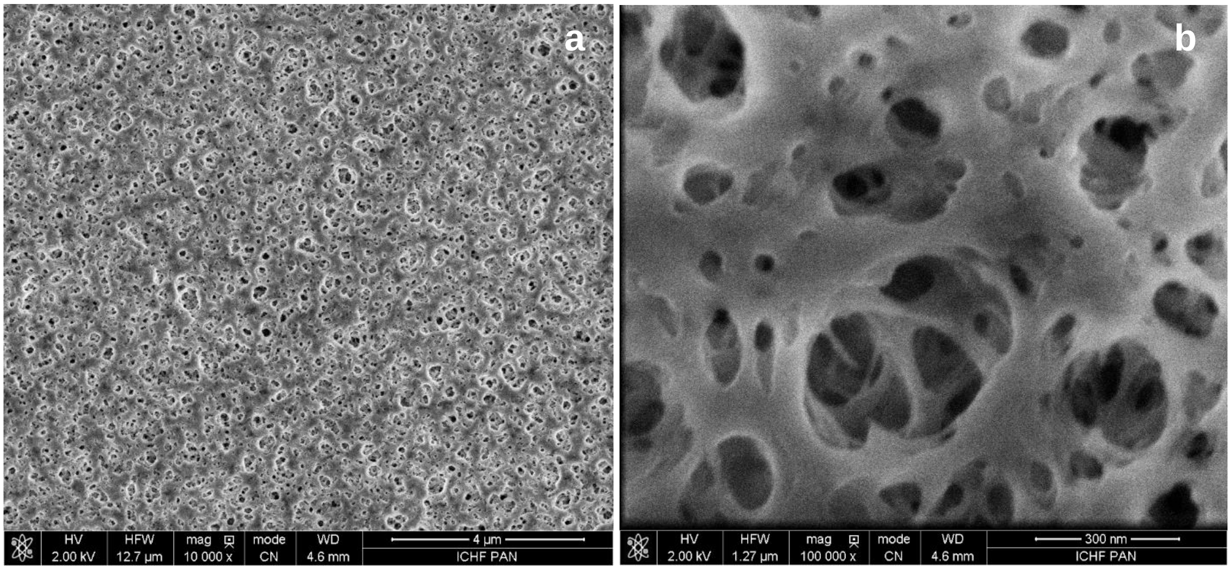

Figure S2. Different magnification SEM images of a microgel prepared using a solution of 25 mM MA, 25 mM NIPAM, 50 mM BIS, 25 mM APS, and 0.1 M in KNO_3, pH = 7.4.

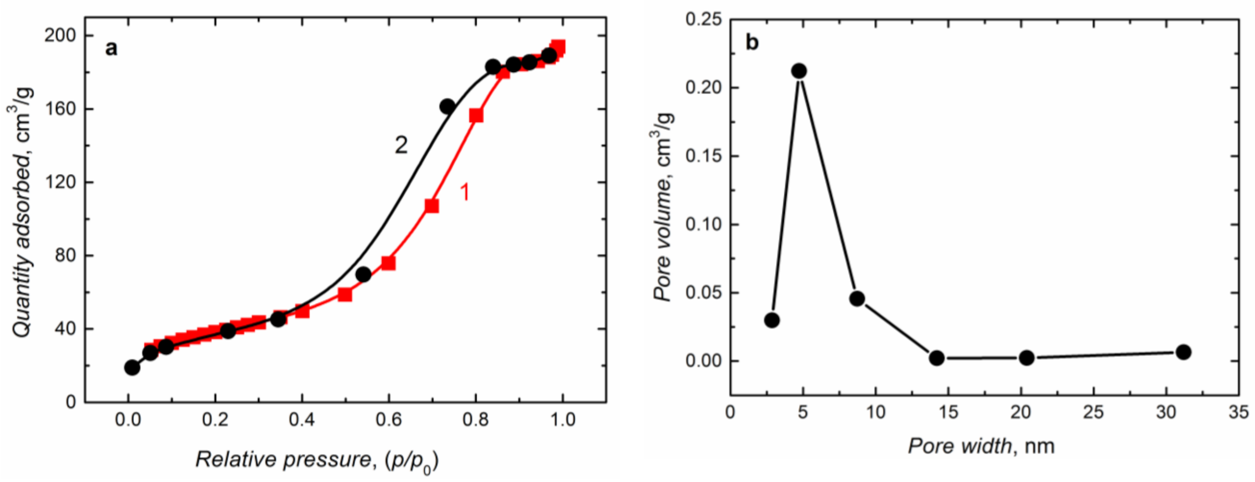

Figure S3. (a) The BET isotherm of N_2 (curve 1) adsorption and (curve 2) desorption for the microgel prepared by electrochemically initiated polymerization of NIPAM**,** MA, and BIS, and (b) the pore width distribution for the resulting microgel particles.

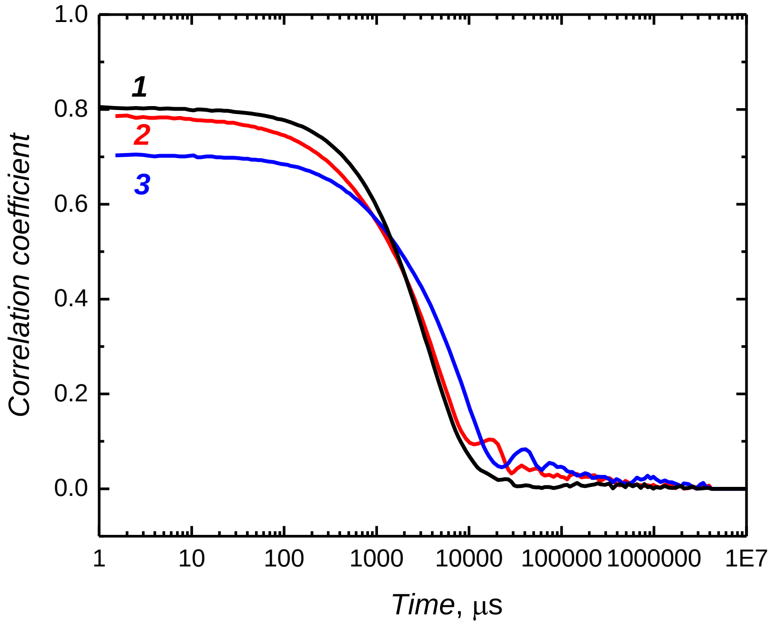

Figure S4. The correlation function intensity for three microgel particles prepared under hydrodynamic conditions by electrochemically initiated polymerization of the (1) NIPAM-MA-BIS, (2) NIPAM-BIS, and (3) NIPAM-MA monomer combinations.

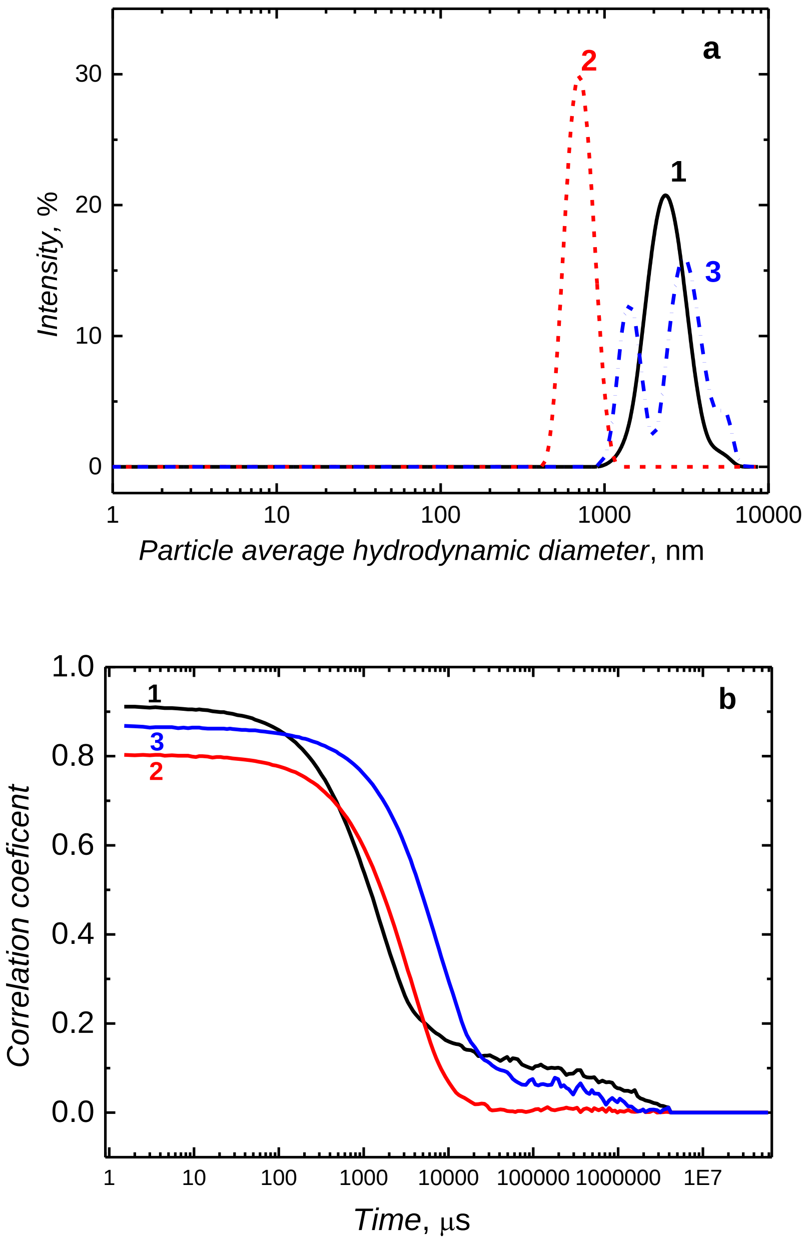

Figure S5. (a) The microgel hydrodynamic particle size, DLS measured during electrochemically initiated polymerization under hydrodynamic conditions at (curve 1) 0, (curve 2) 30 min, and (curve 3) 1 h after initiating the polymerization. (b) The correlation function intensity for (curve 1) 0, (curve 2) 30 min, and (curve 3) 1 h after initiating the polymerization. The microgel was prepared by combining NIPAM, MA, and BIS monomers.

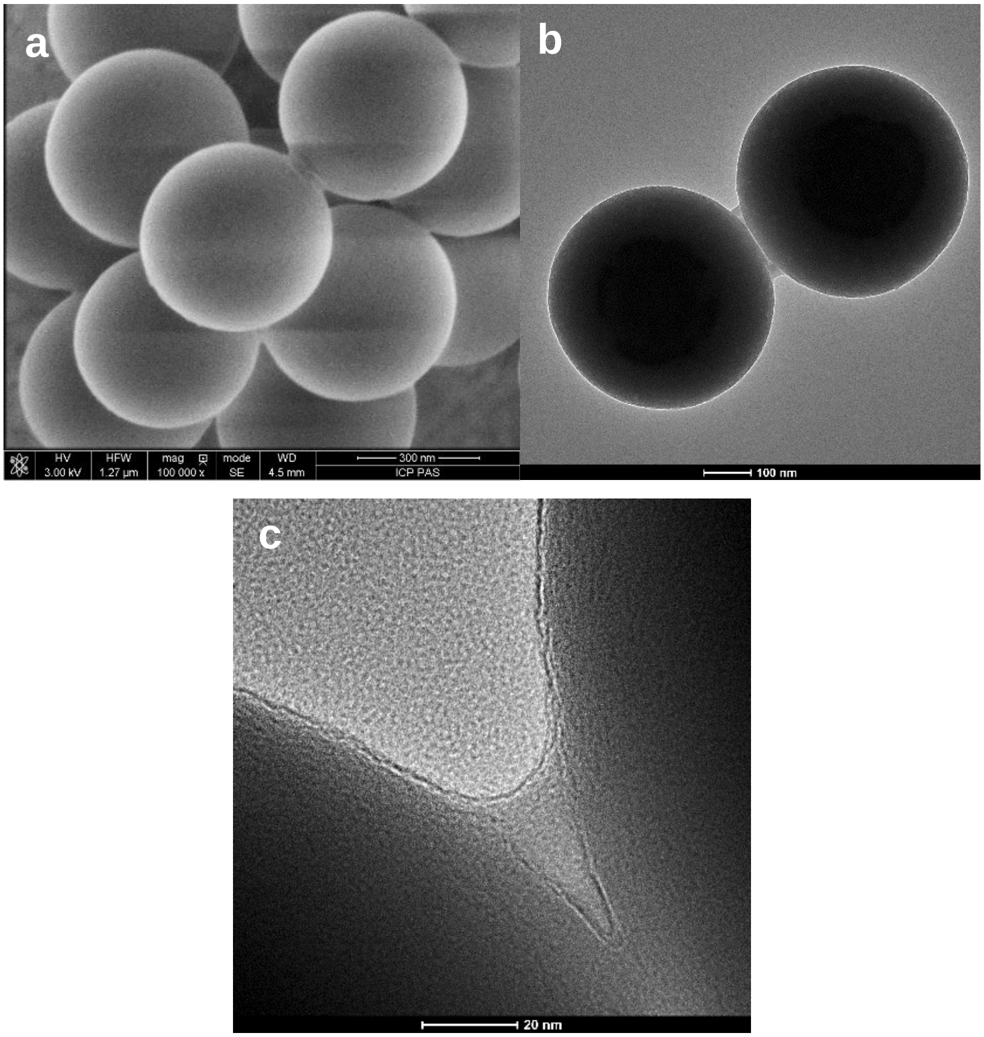

Figure S6. The (a) SEM and (b, c) STEM images of the (silica nanoparticle)-(NIPAM-MA) core-shell beads.
